# 4-Methyl-*N*-{2-[(*E*)-3-oxo-3-phenyl­prop-1-en-1-yl]phen­yl}benzene­sulfonamide

**DOI:** 10.1107/S1600536814015311

**Published:** 2014-07-05

**Authors:** Sung-Gon Kim

**Affiliations:** a Department of Chemistry, Kyonggi University, 154-42, Gwanggyosan-ro, Yeongtong-gu, Suwon 443-760, Republic of Korea

**Keywords:** crystal structure

## Abstract

In the title compound, C_22_H_19_NO_3_S, the terminal phenyl and methyl­phenyl rings are twisted by 37.35 (12) and 49.08 (13)°, respectively, to the central benzene ring. In the crystal, mol­ecules are linked by classical N—H⋯O hydrogen bonds and weak C—H⋯O hydrogen bonds into a three-dimensional supra­molecular network.

## Related literature   

For applications of sulfonamides in the fields of chemistry, biology and pharmacology, see: Chohan *et al.* (2010 ▶); El-Sayed *et al.* (2011 ▶); Seri *et al.* (2000 ▶); Suparan *et al.* (2000 ▶). For related structures, see: Murugavel *et al.* (2012 ▶); Zhang *et al.* (2010 ▶); Mughal *et al.* (2012 ▶).
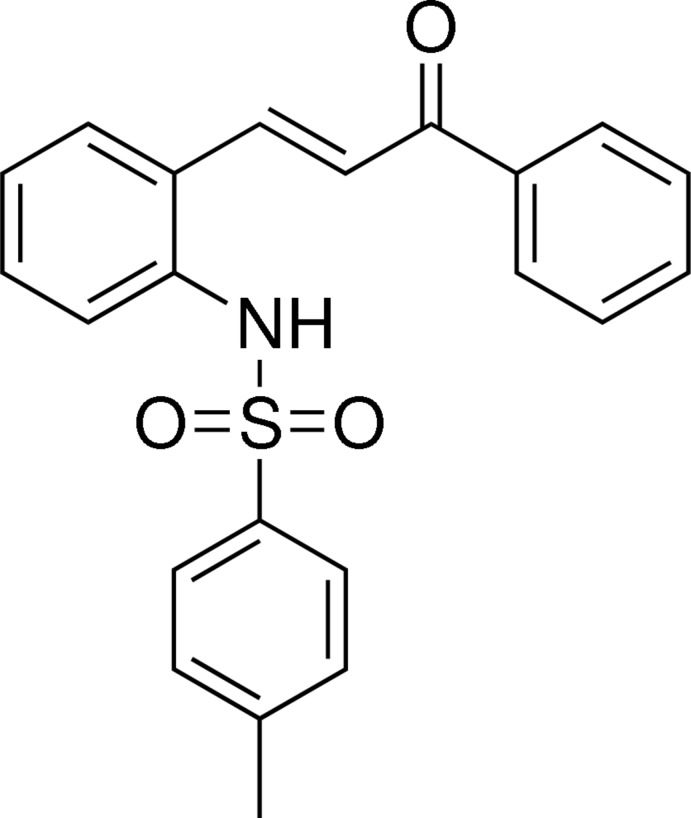


## Experimental   

### 

#### Crystal data  

C_22_H_19_NO_3_S
*M* *_r_* = 377.44
Monoclinic, 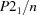

*a* = 8.9356 (7) Å
*b* = 10.8873 (8) Å
*c* = 19.3122 (14) Å
β = 99.472 (2)°
*V* = 1853.2 (2) Å^3^
*Z* = 4
Mo *K*α radiation
μ = 0.20 mm^−1^
*T* = 200 K
0.19 × 0.12 × 0.06 mm

#### Data collection  

Bruker SMART 1000 CCD area-detector diffractometer
Absorption correction: multi-scan (*SADABS*; Bruker, 2001 ▶) *T*_min_ = 0.964, *T*_max_ = 0.988
13392 measured reflections
4589 independent reflections
2366 reflections with *I* > 2σ(*I*)
*R*_int_ = 0.066

#### Refinement  

*R*[*F* ^2^ > 2σ(*F* ^2^)] = 0.057
*wR*(*F* ^2^) = 0.154
*S* = 1.01
4589 reflections
245 parameters
H-atom parameters constrained
Δρ_max_ = 0.46 e Å^−3^
Δρ_min_ = −0.43 e Å^−3^

### 

Data collection: *SMART* (Bruker, 2007 ▶); cell refinement: *SAINT* (Bruker, 2007 ▶); data reduction: *SAINT*; program(s) used to solve structure: *SHELXTL* (Sheldrick, 2008 ▶); program(s) used to refine structure: *SHELXTL*; molecular graphics: *ORTEP-3 for Windows* (Farrugia, 2012 ▶); software used to prepare material for publication: *SHELXTL* and *publCIF* (Westrip, 2010 ▶).

## Supplementary Material

Crystal structure: contains datablock(s) I, global. DOI: 10.1107/S1600536814015311/xu5798sup1.cif

Structure factors: contains datablock(s) I. DOI: 10.1107/S1600536814015311/xu5798Isup2.hkl

Supporting information file. DOI: 10.1107/S1600536814015311/xu5798Isup3.cml

CCDC reference: 1010977

Additional supporting information:  crystallographic information; 3D view; checkCIF report

## Figures and Tables

**Table 1:** Hydrogen-bond geometry (Å, °)

*D*—H⋯*A*	*D*—H	H⋯*A*	*D*⋯*A*	*D*—H⋯*A*
N1—H1⋯O1^i^	0.88	2.24	2.839 (3)	125
C4—H4⋯O2^i^	0.95	2.51	3.272 (4)	137
C18—H18⋯O3^ii^	0.95	2.44	3.373 (3)	167
C22—H22*B*⋯O1^iii^	0.98	2.58	3.466 (4)	150

Symmetry codes: (i) 
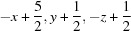
; (ii) 
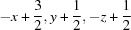
; (iii) 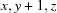.

**Fig. 1.**
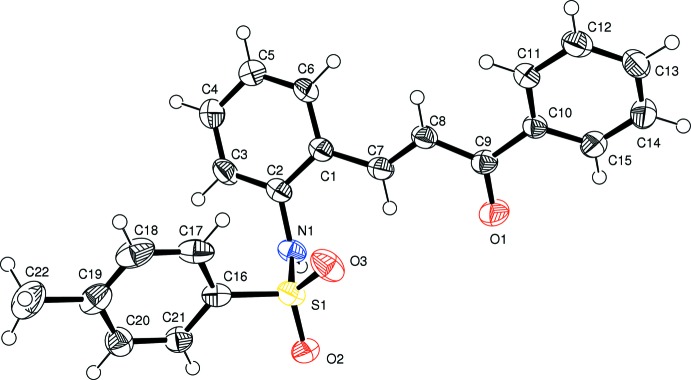
A view of the molecular structure of the title compound, showing the atom-numbering scheme. Displacement ellipsoids are drawn at the 50% probability level.

**Fig. 2.**
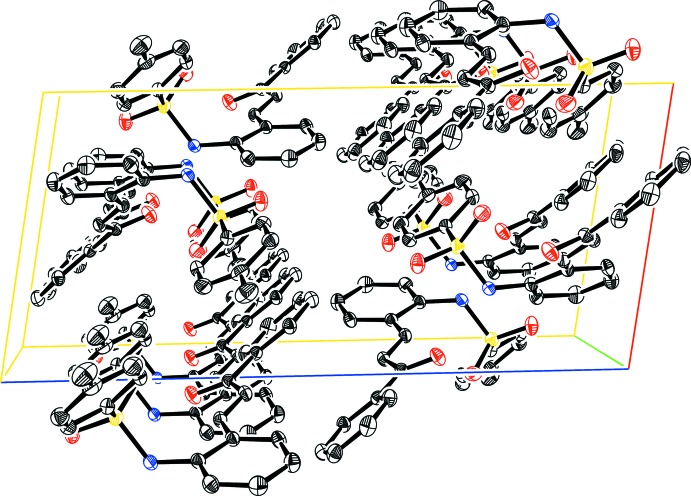
A partial view of the crystal packing of the title compound. Hydrogen atoms have been omitted for clarity.

## supplementary crystallographic information

### S2. Structural commentary

For related structures, see: Murugavel *et al.* (2012); Zhang *et al.* (2010). Sulfonamides, which are already known as sulfa drugs, are an important class of compounds in the field of chemistry, biology and pharmacology. Several sulfonamide derivatives are used as chemotherapeutic agents for their anti­bacterial, anti­fungal, anti­tumor and hypoglycemic (Chohan *et al.*, 2010; El-Sayed *et al.*, 2011; Seri *et al.*, 2000). In addition, some sulfonamide derivatives have been shown to inhibit on carbonic anhydrases (Suparan *et al.*, 2000). In view of these potential applications and in continuation of our work, the structure of the title compound has been carried out and the results are presented here.

X-ray analysis confirms the molecular structure and atom connectivity as illustrated in Fig. 1. The geometry around atoms S and N are distorted tetra­hedral and planar trigonal, respectively. The average S—O bond length is 1.427 (2) Å, while the S—N and S—C bond lengths are 1.638 (3) and 1.757 (3), respectively. The dihedral angle between the vinyl­phenyl ring (C1—C6) and the methyl­phenyl ring (C16—C21) is 49.08 (13)^o^, and the dihedral angle between the vinyl­phenyl ring (C1—C6) and keto­phenyl (C10—C15) ring is 37.35 (12)^o^. The sulfonamide torsion angle 1s 64.0 (2)^o^ for C2—N1—S1—C16. The C==C double bond is in an *E* conformation and the vinyl­carbonyl groups adopt extended conformations.

### S5. Synthesis and crystallization

A solution of 4 M Na_2_CO_3_ in water (10 mL) was added to a solution of 2-amino­benzyl alcohol (5.0 mmol) and *p*-toluene­sulfonyl chloride (5.5 mmol) in THF (10 mL). After stirring at room temperature for 24 h, the reaction mixture was poured into cold water and extracted with EtOAc. The resultant organic layer was washed with brine and dried over MgSO_4_. The resulting residue was purified by silica gel chromatography to afford 2-(toluensulfonyl­amino)­benzyl alcohol. Next, to solutionof 2-(toluensulfonyl­amino)­benzyl alcohol (3.0 mmol) in CH_2_Cl_2_ (10 mL) was added excess MnO_2_ (15 mmol). After stirring for at room temperature for 36 h, the reaction mixture was filtered under celite pad and purified by silica gel chromatography to afford 2-(toluensulfonyl­amino)­benzaldehyde. To a solution of phenacyl­tri­phenyl­phospho­nium bromide (1.1 mmol) in toluene (5 mL) was added NaH (1.2 mmol) at 273 K. After stirring at 273 K for 1 h, to the reactin mixture, 2-(toluensulfonyl­amino)­benzaldehyde (1.0 mmol) was added. After stirring at room temperature for 24 h, the reaction mixture was poured into water and extracted with EtOAc. The resultant organic layer was washed with brine and dried over MgSO_4_. Theresulting residue was purified by silica gel chromatography to afford the title compound. Crystals suitable for X-ray analysis were obtained by recryatallization from an n-hexane/CH_2_Cl_2_ solution.

### S6. Refinement

All H atoms were positioned geometrically, (C—H = 0.95–0.96 Å) and constrained to ride on their parent atoms, with *U*_iso_(H) = *xU*eq(C), where *x* = 1.2 for all other H atoms.

### Crystal data

**Table:**

C_22_H_19_NO_3_S	*F*(000) = 792
*M**_r_* = 377.44	*D*_x_ = 1.353 Mg m^−^^3^
Monoclinic, *P*2_1_/*n*	Mo *K*α radiation, λ = 0.71073 Å
Hall symbol: -P 2yn	Cell parameters from 2689 reflections
*a* = 8.9356 (7) Å	θ = 2.4–26.1°
*b* = 10.8873 (8) Å	µ = 0.20 mm^−^^1^
*c* = 19.3122 (14) Å	*T* = 200 K
β = 99.472 (2)°	Rod, colorless
*V* = 1853.2 (2) Å^3^	0.19 × 0.12 × 0.06 mm
*Z* = 4	

### Data collection

**Table:**

Bruker SMART 1000 CCD area-detector diffractometer	4589 independent reflections
Radiation source: fine-focus sealed tube	2366 reflections with *I* > 2σ(*I*)
Graphite monochromator	*R*_int_ = 0.066
phi and ω scans	θ_max_ = 28.3°, θ_min_ = 2.1°
Absorption correction: multi-scan (*SADABS*; Bruker, 2001)	*h* = −11→11
*T*_min_ = 0.964, *T*_max_ = 0.988	*k* = −14→13
13392 measured reflections	*l* = −25→24

### Refinement

**Table:**

Refinement on *F*^2^	Primary atom site location: structure-invariant direct methods
Least-squares matrix: full	Secondary atom site location: difference Fourier map
*R*[*F*^2^ > 2σ(*F*^2^)] = 0.057	Hydrogen site location: inferred from neighbouring sites
*wR*(*F*^2^) = 0.154	H-atom parameters constrained
*S* = 1.01	*w* = 1/[σ^2^(*F*_o_^2^) + (0.0583*P*)^2^] where *P* = (*F*_o_^2^ + 2*F*_c_^2^)/3
4589 reflections	(Δ/σ)_max_ < 0.001
245 parameters	Δρ_max_ = 0.46 e Å^−^^3^
0 restraints	Δρ_min_ = −0.43 e Å^−^^3^

### Special details

**Table:**

Geometry. All e.s.d.'s (except the e.s.d. in the dihedral angle between two l.s. planes) are estimated using the full covariance matrix. The cell e.s.d.'s are taken into account individually in the estimation of e.s.d.'s in distances, angles and torsion angles; correlations between e.s.d.'s in cell parameters are only used when they are defined by crystal symmetry. An approximate (isotropic) treatment of cell e.s.d.'s is used for estimating e.s.d.'s involving l.s. planes.
Refinement. Refinement of *F*^2^ against ALL reflections. The weighted *R*-factor *wR* and goodness of fit *S* are based on *F*^2^, conventional *R*-factors *R* are based on *F*, with *F* set to zero for negative *F*^2^. The threshold expression of *F*^2^ > σ(*F*^2^) is used only for calculating *R*-factors(gt) *etc*. and is not relevant to the choice of reflections for refinement. *R*-factors based on *F*^2^ are statistically about twice as large as those based on *F*, and *R*- factors based on ALL data will be even larger.

### Fractional atomic coordinates and isotropic or equivalent isotropic displacement parameters (Å^2^)

**Table:**

	*x*	*y*	*z*	*U*_iso_*/*U*_eq_	
C1	1.1405 (3)	0.4390 (2)	0.37034 (13)	0.0321 (6)	
C2	1.1969 (3)	0.5180 (3)	0.32288 (13)	0.0340 (6)	
C3	1.2509 (3)	0.6334 (3)	0.34430 (14)	0.0407 (7)	
H3	1.2877	0.6861	0.3117	0.049*	
C4	1.2520 (3)	0.6731 (3)	0.41228 (15)	0.0459 (8)	
H4	1.2912	0.7518	0.4267	0.055*	
C5	1.1952 (3)	0.5965 (3)	0.45938 (15)	0.0439 (8)	
H5	1.1947	0.6233	0.5062	0.053*	
C6	1.1398 (3)	0.4822 (3)	0.43849 (13)	0.0372 (7)	
H6	1.1001	0.4314	0.4711	0.045*	
C7	1.0964 (3)	0.3133 (2)	0.35017 (13)	0.0336 (6)	
H7	1.1236	0.2853	0.3073	0.040*	
C8	1.0229 (3)	0.2331 (2)	0.38424 (13)	0.0335 (6)	
H8	0.9856	0.2581	0.4253	0.040*	
C9	0.9987 (3)	0.1067 (2)	0.35913 (14)	0.0326 (6)	
O1	1.0479 (2)	0.07285 (18)	0.30617 (10)	0.0461 (5)	
C10	0.9149 (3)	0.0178 (2)	0.39676 (13)	0.0300 (6)	
C11	0.8184 (3)	0.0538 (3)	0.44267 (13)	0.0355 (7)	
H11	0.8081	0.1386	0.4526	0.043*	
C12	0.7373 (3)	−0.0321 (3)	0.47403 (14)	0.0426 (7)	
H12	0.6711	−0.0066	0.5050	0.051*	
C13	0.7536 (4)	−0.1553 (3)	0.45992 (14)	0.0470 (8)	
H13	0.6968	−0.2146	0.4807	0.056*	
C14	0.8511 (4)	−0.1927 (3)	0.41609 (15)	0.0493 (8)	
H14	0.8632	−0.2778	0.4076	0.059*	
C15	0.9315 (3)	−0.1073 (3)	0.38437 (14)	0.0400 (7)	
H15	0.9986	−0.1337	0.3540	0.048*	
N1	1.2050 (2)	0.4787 (2)	0.25294 (10)	0.0350 (6)	
H1	1.2927	0.4543	0.2427	0.042*	
S1	1.05508 (8)	0.47777 (7)	0.19146 (4)	0.0388 (2)	
O2	1.1089 (2)	0.43544 (17)	0.13014 (9)	0.0465 (5)	
O3	0.9384 (2)	0.41316 (19)	0.21857 (10)	0.0520 (6)	
C16	0.9960 (3)	0.6311 (3)	0.17777 (13)	0.0359 (7)	
C17	0.8957 (3)	0.6821 (3)	0.21770 (14)	0.0464 (8)	
H17	0.8601	0.6346	0.2529	0.056*	
C18	0.8480 (3)	0.8018 (3)	0.20592 (16)	0.0533 (9)	
H18	0.7802	0.8365	0.2336	0.064*	
C19	0.8975 (3)	0.8729 (3)	0.15412 (16)	0.0455 (8)	
C20	0.9965 (3)	0.8202 (3)	0.11462 (15)	0.0423 (7)	
H20	1.0310	0.8672	0.0789	0.051*	
C21	1.0464 (3)	0.7003 (3)	0.12619 (14)	0.0360 (7)	
H21	1.1150	0.6658	0.0988	0.043*	
C22	0.8426 (4)	1.0035 (3)	0.14254 (18)	0.0638 (10)	
H22A	0.8766	1.0368	0.1006	0.096*	
H22B	0.8840	1.0535	0.1834	0.096*	
H22C	0.7315	1.0051	0.1361	0.096*	

### Atomic displacement parameters (Å^2^)

**Table:**

	*U*^11^	*U*^22^	*U*^33^	*U*^12^	*U*^13^	*U*^23^
C1	0.0322 (15)	0.0345 (16)	0.0305 (14)	0.0012 (12)	0.0077 (12)	0.0040 (12)
C2	0.0333 (16)	0.0384 (17)	0.0312 (14)	0.0015 (13)	0.0079 (12)	0.0065 (13)
C3	0.0464 (18)	0.0390 (18)	0.0379 (16)	−0.0060 (14)	0.0108 (14)	0.0105 (14)
C4	0.054 (2)	0.0364 (18)	0.0481 (18)	−0.0060 (15)	0.0094 (15)	0.0004 (15)
C5	0.054 (2)	0.0407 (18)	0.0376 (16)	−0.0011 (15)	0.0102 (14)	−0.0026 (14)
C6	0.0447 (18)	0.0358 (16)	0.0336 (15)	−0.0023 (14)	0.0144 (13)	0.0054 (13)
C7	0.0361 (16)	0.0382 (17)	0.0275 (14)	0.0048 (13)	0.0078 (12)	0.0029 (12)
C8	0.0359 (16)	0.0367 (16)	0.0296 (14)	0.0006 (12)	0.0103 (12)	0.0025 (12)
C9	0.0310 (15)	0.0357 (16)	0.0321 (14)	0.0045 (12)	0.0081 (12)	−0.0012 (13)
O1	0.0521 (13)	0.0464 (13)	0.0453 (12)	0.0009 (10)	0.0245 (10)	−0.0084 (10)
C10	0.0316 (15)	0.0308 (15)	0.0268 (13)	−0.0004 (12)	0.0024 (11)	0.0014 (12)
C11	0.0415 (17)	0.0350 (17)	0.0310 (14)	−0.0024 (13)	0.0085 (13)	0.0031 (12)
C12	0.0448 (18)	0.050 (2)	0.0348 (15)	−0.0050 (15)	0.0106 (13)	0.0025 (15)
C13	0.061 (2)	0.044 (2)	0.0353 (16)	−0.0149 (16)	0.0037 (15)	0.0074 (15)
C14	0.069 (2)	0.0337 (18)	0.0433 (18)	−0.0035 (16)	0.0036 (16)	0.0000 (14)
C15	0.0487 (19)	0.0356 (17)	0.0356 (15)	0.0025 (14)	0.0065 (14)	−0.0013 (13)
N1	0.0308 (13)	0.0458 (14)	0.0300 (11)	0.0047 (11)	0.0092 (10)	0.0054 (11)
S1	0.0408 (4)	0.0434 (5)	0.0324 (4)	−0.0028 (3)	0.0070 (3)	0.0039 (3)
O2	0.0667 (15)	0.0398 (12)	0.0338 (10)	0.0078 (10)	0.0112 (10)	−0.0038 (9)
O3	0.0428 (13)	0.0633 (15)	0.0489 (12)	−0.0159 (11)	0.0043 (10)	0.0154 (11)
C16	0.0346 (16)	0.0450 (18)	0.0279 (14)	−0.0004 (13)	0.0047 (12)	−0.0019 (13)
C17	0.0419 (18)	0.069 (2)	0.0302 (15)	0.0050 (16)	0.0111 (13)	−0.0014 (15)
C18	0.0402 (19)	0.072 (3)	0.0471 (19)	0.0184 (17)	0.0051 (15)	−0.0167 (18)
C19	0.0378 (18)	0.050 (2)	0.0449 (18)	0.0093 (15)	−0.0058 (14)	−0.0130 (16)
C20	0.0419 (18)	0.0417 (18)	0.0423 (17)	0.0013 (14)	0.0039 (14)	0.0037 (14)
C21	0.0328 (16)	0.0405 (17)	0.0355 (15)	0.0025 (13)	0.0078 (12)	−0.0018 (13)
C22	0.062 (2)	0.056 (2)	0.068 (2)	0.0236 (18)	−0.0084 (18)	−0.0161 (19)

### Geometric parameters (Å, º)

**Table:**

C1—C6	1.399 (3)	C13—C14	1.373 (4)
C1—C2	1.409 (3)	C13—H13	0.9500
C1—C7	1.459 (4)	C14—C15	1.379 (4)
C2—C3	1.384 (4)	C14—H14	0.9500
C2—N1	1.430 (3)	C15—H15	0.9500
C3—C4	1.380 (4)	N1—S1	1.638 (2)
C3—H3	0.9500	N1—H1	0.8800
C4—C5	1.390 (4)	S1—O2	1.4259 (19)
C4—H4	0.9500	S1—O3	1.427 (2)
C5—C6	1.375 (4)	S1—C16	1.757 (3)
C5—H5	0.9500	C16—C21	1.382 (4)
C6—H6	0.9500	C16—C17	1.391 (4)
C7—C8	1.330 (3)	C17—C18	1.379 (4)
C7—H7	0.9500	C17—H17	0.9500
C8—C9	1.463 (4)	C18—C19	1.393 (4)
C8—H8	0.9500	C18—H18	0.9500
C9—O1	1.234 (3)	C19—C20	1.384 (4)
C9—C10	1.484 (4)	C19—C22	1.509 (4)
C10—C11	1.391 (4)	C20—C21	1.386 (4)
C10—C15	1.395 (4)	C20—H20	0.9500
C11—C12	1.383 (4)	C21—H21	0.9500
C11—H11	0.9500	C22—H22A	0.9800
C12—C13	1.381 (4)	C22—H22B	0.9800
C12—H12	0.9500	C22—H22C	0.9800
			
C6—C1—C2	117.7 (2)	C13—C14—C15	120.3 (3)
C6—C1—C7	121.6 (2)	C13—C14—H14	119.9
C2—C1—C7	120.6 (2)	C15—C14—H14	119.9
C3—C2—C1	120.3 (2)	C14—C15—C10	120.3 (3)
C3—C2—N1	119.0 (2)	C14—C15—H15	119.9
C1—C2—N1	120.7 (2)	C10—C15—H15	119.9
C4—C3—C2	121.1 (3)	C2—N1—S1	121.59 (18)
C4—C3—H3	119.5	C2—N1—H1	119.2
C2—C3—H3	119.5	S1—N1—H1	119.2
C3—C4—C5	119.2 (3)	O2—S1—O3	120.81 (13)
C3—C4—H4	120.4	O2—S1—N1	104.87 (12)
C5—C4—H4	120.4	O3—S1—N1	107.27 (12)
C6—C5—C4	120.2 (3)	O2—S1—C16	108.42 (12)
C6—C5—H5	119.9	O3—S1—C16	107.69 (13)
C4—C5—H5	119.9	N1—S1—C16	107.05 (12)
C5—C6—C1	121.5 (3)	C21—C16—C17	119.9 (3)
C5—C6—H6	119.2	C21—C16—S1	120.1 (2)
C1—C6—H6	119.2	C17—C16—S1	120.1 (2)
C8—C7—C1	128.1 (2)	C18—C17—C16	119.7 (3)
C8—C7—H7	115.9	C18—C17—H17	120.1
C1—C7—H7	115.9	C16—C17—H17	120.1
C7—C8—C9	120.8 (2)	C17—C18—C19	121.2 (3)
C7—C8—H8	119.6	C17—C18—H18	119.4
C9—C8—H8	119.6	C19—C18—H18	119.4
O1—C9—C8	120.1 (2)	C20—C19—C18	118.2 (3)
O1—C9—C10	119.2 (2)	C20—C19—C22	122.0 (3)
C8—C9—C10	120.6 (2)	C18—C19—C22	119.7 (3)
C11—C10—C15	118.6 (2)	C19—C20—C21	121.3 (3)
C11—C10—C9	123.0 (2)	C19—C20—H20	119.4
C15—C10—C9	118.4 (2)	C21—C20—H20	119.4
C12—C11—C10	120.9 (3)	C16—C21—C20	119.7 (3)
C12—C11—H11	119.6	C16—C21—H21	120.1
C10—C11—H11	119.6	C20—C21—H21	120.1
C13—C12—C11	119.4 (3)	C19—C22—H22A	109.5
C13—C12—H12	120.3	C19—C22—H22B	109.5
C11—C12—H12	120.3	H22A—C22—H22B	109.5
C14—C13—C12	120.5 (3)	C19—C22—H22C	109.5
C14—C13—H13	119.8	H22A—C22—H22C	109.5
C12—C13—H13	119.8	H22B—C22—H22C	109.5
			
C6—C1—C2—C3	0.8 (4)	C13—C14—C15—C10	0.2 (4)
C7—C1—C2—C3	−174.3 (3)	C11—C10—C15—C14	1.3 (4)
C6—C1—C2—N1	178.2 (2)	C9—C10—C15—C14	−177.4 (3)
C7—C1—C2—N1	3.1 (4)	C3—C2—N1—S1	−103.3 (3)
C1—C2—C3—C4	0.6 (4)	C1—C2—N1—S1	79.2 (3)
N1—C2—C3—C4	−176.9 (3)	C2—N1—S1—O2	179.0 (2)
C2—C3—C4—C5	−1.2 (4)	C2—N1—S1—O3	−51.4 (2)
C3—C4—C5—C6	0.5 (5)	C2—N1—S1—C16	64.0 (2)
C4—C5—C6—C1	0.8 (5)	O2—S1—C16—C21	−19.3 (3)
C2—C1—C6—C5	−1.5 (4)	O3—S1—C16—C21	−151.6 (2)
C7—C1—C6—C5	173.6 (3)	N1—S1—C16—C21	93.3 (2)
C6—C1—C7—C8	15.1 (4)	O2—S1—C16—C17	159.2 (2)
C2—C1—C7—C8	−170.0 (3)	O3—S1—C16—C17	26.9 (3)
C1—C7—C8—C9	−174.9 (2)	N1—S1—C16—C17	−88.2 (2)
C7—C8—C9—O1	0.7 (4)	C21—C16—C17—C18	−0.5 (4)
C7—C8—C9—C10	−179.2 (2)	S1—C16—C17—C18	−179.0 (2)
O1—C9—C10—C11	−160.3 (2)	C16—C17—C18—C19	0.6 (5)
C8—C9—C10—C11	19.6 (4)	C17—C18—C19—C20	−0.2 (4)
O1—C9—C10—C15	18.4 (4)	C17—C18—C19—C22	179.8 (3)
C8—C9—C10—C15	−161.7 (2)	C18—C19—C20—C21	−0.3 (4)
C15—C10—C11—C12	−1.7 (4)	C22—C19—C20—C21	179.7 (3)
C9—C10—C11—C12	177.0 (2)	C17—C16—C21—C20	0.0 (4)
C10—C11—C12—C13	0.5 (4)	S1—C16—C21—C20	178.5 (2)
C11—C12—C13—C14	1.1 (4)	C19—C20—C21—C16	0.4 (4)
C12—C13—C14—C15	−1.5 (4)		

### Hydrogen-bond geometry (Å, º)

**Table:**

*D*—H···*A*	*D*—H	H···*A*	*D*···*A*	*D*—H···*A*
N1—H1···O1^i^	0.88	2.24	2.839 (3)	125
C4—H4···O2^i^	0.95	2.51	3.272 (4)	137
C18—H18···O3^ii^	0.95	2.44	3.373 (3)	167
C22—H22*B*···O1^iii^	0.98	2.58	3.466 (4)	150

Symmetry codes: (i) −*x*+5/2, *y*+1/2, −*z*+1/2; (ii) −*x*+3/2, *y*+1/2, −*z*+1/2; (iii) *x*, *y*+1, *z*.
